# Astrocyte Reactivity and White Matter Integrity in the Context of Alzheimer’s Disease Pathologies

**DOI:** 10.1002/alz.084224

**Published:** 2025-01-09

**Authors:** Oceanna Yueran Li, Adam Turnbull, Feng Vankee Lin

**Affiliations:** ^1^ Stanford University, Stanford, CA USA

## Abstract

**Background:**

Astrocytes that become reactive in response to damage (e.g., due to typical brain aging or Alzheimer’s Disease/AD pathologies) can have both adaptive (e.g., maintaining myelination and clearing debris) and maladaptive (e.g., facilitating the spread of AD pathologies) functions. Here, we investigate how two protein markers of astrocyte reactivity (glial fibrillary acidic protein/GFAP and chitinase‐3‐like protein 1/YKL‐40) relate to medial temporal lobe (MTL) WM integrity and episodic memory in older adults with and without AD pathologies. We hypothesize that astrocyte reactivity would maintain WM stability and support memory when AD pathologies are absent.

**Method:**

Participants with at least two repeated DTI sessions, 2 years apart, and cerebrospinal fluid (CSF) proteomics data were selected from the Alzheimer's Disease Neuroimaging Initiative (ADNI) GO/2 sample (n=56). We selected two CSF proteomics biomarkers of astrocyte reactivity based on previous meta‐analyses – GFAP and YKL‐40. WM stability in MTL networks was quantified via correlation coefficients of mean diffusivity between baseline and 2‐year follow‐up. CSF‐based beta‐amyloid and p‐tau at baseline were also included. A mean episodic memory composite was calculated across both timepoints.

**Result:**

Higher GFAP and lower YKL‐40 levels at baseline were related to higher stability of MTL networks. The relationship between GFAP, but not YKL‐40, was moderated by beta‐amyloid pathology. When beta‐amyloid was absent, higher GFAP was related to greater WM stability and memory; higher WM stability mediated links between higher GFAP and better mean memory. When beta‐amyloid was present, higher GFAP was related to worse p‐tau but not to WM stability.

**Conclusion:**

Our study examined the relationships between two protein markers of astrocyte reactivity, AD pathology, and MTL network WM stability. We replicated the interaction GFAP and beta‐amyloid in predicting p‐tau that implicates reactive astrocytes in the beta‐amyloid dependent propagation of p‐tau. The correlation between GFAP levels and both WM stability and memory in the absence of beta‐amyloid suggests astrocytic reactivity in healthy aging may contribute to the maintenance of WM connectivity. Alternatively, higher YKL‐40 was related to worse WM stability regardless of AD pathology suggests that YKL‐40 may reflect a reactive astrocyte state that is detrimental in healthy aging.

